# Impact of gang violence in Haiti on healthcare delivery and medical education

**DOI:** 10.1016/j.lana.2024.100811

**Published:** 2024-06-08

**Authors:** Arens Jean Ricardo Medeus, Claudy Junior Pierre, Ludjie Love Smeischelle Merilan, Sterline Vertilus

**Affiliations:** a Department of General Surgery, State University Hospital of Haiti (HUEH), Ave.Mgr Guilloux et St-Honoré, Port-au-Prince, Haiti; b Surgical Research and Global Education Lab (Surge Lab), Port-au-Prince, Haiti; c Faculty of Medicine and Pharmacy of the State University of Haiti (FMP/UEH), Rue Oswald Durand, Port-au-Prince, Haiti

Haiti, a Caribbean island nation, faces a severe crisis exacerbated by gang violence, chronic political turmoil, and recurring natural disasters. The capital, Port-au-Prince, has become a battleground for rival gangs seeking dominance. A coalition of eight powerful gangs terrorizes the population with massacres, looting, and arson, targeting schools, universities, and hospitals. This escalation of violence threatens the security and well-being of Haitians and destabilizes the socio-economic fabric of the country.^1^

The impact of gang violence on Haitian society is profound, particularly in healthcare delivery and medical education. The State University Hospital of Haiti, the nation's largest university hospital, and Saint-François de Sales Hospital, one of the few with an oncology department, have been targeted.^2^^,^^3^ In March 2024, these hospitals, serving over 500,000 residents, were vandalized and looted.^4^ Dr. Bernard Joseph Jr reported that solar panels and computer equipment were stolen from Saint-François-de-Sales Hospital.^5^ The State University Hospital has been under gang control since April 1, 2024, with its administrative buildings vandalized.^3^^,^^6^

The closure of these hospitals has left many without access to essential healthcare. Vandalism has also impacted over twenty pharmacies, ten pharmaceutical depots, two agencies, and a laboratory. The health directorate of the West reported that between February 29 and March 21, 2024, eighteen healthcare institutions became non-functional, including major hospitals in the metropolitan area. Croix-des-Bouquets, the largest municipality, has only three functional hospitals as of April 11, 2024. The closure of the port and airport since March 5, 2024, has led to a nationwide shortage of medical supplies.

The Association of Private Hospitals of Haiti (AHPH) expressed deep concern over the critical situation hindering their ability to provide quality care, especially for gunshot victims. The pervasive insecurity has forced doctors to reduce their private practice and implement safety measures like call screening and remote consultations, though threats persist. Residents and medical students face restricted mobility and increased stress, leading to interrupted clinical rotations and a decrease in the diversity of clinical cases. Safety concerns during rotations in gang-active areas compromise their training and future commitment.

Educators are also affected, with some leaving the country or being too traumatized to teach. Online courses have been proposed as a solution, but this approach faces significant challenges, such as lack of electricity and unstable internet connections. The ongoing territorial war in the capital makes it impossible to return to educational institutions, further disrupting medical education.

In response to the gang violence, the Haitian government has made some attempts to address the crisis. The government promptly declared a state of emergency across the country and implemented a curfew starting at 6 pm., which remained in effect for one month.^7^ In the “Champ-de-Mars” area, the national police used containers to block key streets leading to the city center in an effort to limit the gangs' territorial expansion. The Haitian National Police (PNH) engaged in armed confrontations with the gangs to curb their advances. However, the police force lacks the necessary military resources to effectively combat the criminals and reclaim the occupied territories.

Additionally, with the establishment of the Presidential Council on April 30, 2024, there has been progress in following up on the agreement set by former Prime Minister Ariel Henry for the deployment of the Multinational Security Support Mission (MSSM).^8^ This foreign force, which has been anticipated for over six months, is expected to bolster the national police, aid in regaining control of the city center, and secure the economic zones and main roads of the capital.

Addressing these challenges requires increased awareness of psychological trauma and military training for practitioners' safety. Integrating mental health into medical education is crucial to prepare future doctors for Haiti's realities. A holistic approach is necessary to support healthcare professionals and ensure continuity of care amidst widespread violence.

## Contributors

All authors made significant contributions to the writing of the original draft. Arens Jean Ricardo Medeus served as the project administrator, overseeing the revision, consolidation, and summarization of the article into its final form.

## Declaration of interests

The authors have no conflict of interest.

## References

[1] 96,000 Haïtiens déplacés par les violences entre gangs selon un | IOM Haiti. https://haiti.iom.int/fr/news/96-000-haitiens-deplaces-par-les-violences-entre-gangs-selon-un-rapport-de-loim.

[2] Taylor L. (2024). Haiti: hospitals burn to ground as violence rages. BMJ.

[3] Haiti gangs More than 50000 flee capital after surge in violence. https://www.bbc.com/news/world-latin-america-68722178.

[4] Haïti: quelle est la situation dans la capitale Port-au-Prince en proie à la violence des gangs armés?. https://www.francetvinfo.fr/monde/ameriques/haiti-quelle-est-la-situation-dans-la-capitale-port-au-prince-en-proie-a-la-violence-des-gangs-armes_6415609.html.

[5] Scènes de pillage à l'hôpital Saint-François de Sales. https://lenouvelliste.com/article/247070/scenes-de-pillage-a-lhopital-saint-francois-de-sales.

[6] Haiti health system nears collapse as medicine dwindles, gangs attack hospitals. https://www.voanews.com/a/haiti-health-system-nears-collapse-as-medicine-dwindles-gangs-attack-hospitals-/7582014.html.

[7] Haiti extends state of emergency as intruders break into key port terminal. https://edition.cnn.com/2024/03/07/americas/haiti-port-terminal-violence-intl/index.html.

[8] Heading towards MMSS deployment and efforts to enhance the PNH. https://lenouvelliste.com/en/article/248249/heading-towards-mmss-deployment-and-efforts-to-enhance-the-pnh.

